# Comparative evaluation of microprobe versus conventional transoesophageal echocardiography for PFO closure guidance

**DOI:** 10.1136/openhrt-2023-002502

**Published:** 2024-01-06

**Authors:** Jemina Lanki, Suvi Tuohinen, Piia Simonen, Mikko Jalanko, Juha Sinisalo

**Affiliations:** University of Helsinki, Helsinki, Finland

**Keywords:** Echocardiography, Endovascular Procedures, Heart Septal Defects, Atrial, Stroke

## Abstract

**Background:**

Patent foramen ovale (PFO) closure is traditionally guided by transoesophageal echocardiography (TEE) under general anaesthesia, which prolongs procedure duration and increases costs and risks. A transnasal echocardiography with a microTEE-probe (microTNE) is tolerated under conscious sedation and offers an effective alternative to TEE. The aim of this study was to compare the feasibility, safety and time expenditure of PFO closure using conventional TEE versus microTNE guidance.

**Methods:**

Consecutive patients assigned for PFO losure in Helsinki University Hospital from 2003 to 2021 were included in the study (n=336). TEE with general anaesthesia was used until November 2018 (n=167) while microTNE-guided PFO closure (n=169) under conscious sedation was the principal method thereafter. Patients were followed for 3 months after PFO closure.

**Results:**

The microTNE-route success rate was 97.2% vs TEE 100% (p=0.06) and procedure success rate was 97.7% with microTNE and 96.0% with TEE-guidance (p=0.54). The procedure time was significantly shorter with microTNE 21±7 min than with TEE 30±13 min (p<0.001). At the beginning of microTNE era, nasal bleeding complication was quite frequent; however, overall complication rates were equal between the groups. However, C reactive protein (CRP) increase was significantly milder with microTNE than TEE 1.0±2.9 vs 3.0±4.0 mg/L (p<0.001). An increase in CRP was independently associated with procedure type (p=0.004) and time (p=0.003).

**Conclusions:**

MicroTNE is a feasible and safe alternative for PFO closure guidance. MicroTNE under conscious sedation shortens procedure duration and induces a milder inflammatory reaction than conventional TEE under general anaesthesia.

**WHAT IS ALREADY KNOWN ON THIS TOPIC**

- Conventional transoesophageal echocardiography (TEE) is the main visualisation method for patent foramen ovale (PFO) closure. Transnasal echocardiography with a microTEE-probe (microTNE) is a feasible option; however, no large comparisons between these two methods have been done.

**WHAT THIS STUDY ADDS**

- MicroTNE is a feasible and safe alternative for PFO closure guidance. Under conscious sedation, it shortens procedure duration and induces a milder inflammatory reaction than conventional TEE under general anaesthesia.

**HOW THIS STUDY MIGHT AFFECT RESEARCH, PRACTICE OR POLICY**

- Findings of this study might encourage invasive units to adopt the use of microTNE-guidance for PFO closure. It might reduce the need for already limited anaesthesia resources and increase efficiency.

## Introduction

Patent foramen ovale (PFO) prevalence is approximately 27% globally.^1^ A PFO predisposes to cryptogenic stroke and transient ischaemic attacks (TIAs) by permitting venous thrombi to bypass from the right to the left atrium. Evidence suggests that percutaneous PFO closure is effective in preventing recurrence of thromboembolic events.^2^

The percutaneous PFO closure is a relatively simple procedure. However, general anaesthesia is required when conventional transoesophageal echocardiography (TEE) is used, lengthening the procedure and adding to the risks and costs.^3–6^ Transnasal echocardiography with a microTEE-probe (microTNE) has been found an effective and safe alternative to conventional TEE in diagnostic settings and guiding transcatheter procedures such as aortic or mitral valve implantation or left atrial appendage, atrial septal defect (ASD) or PFO closures.^4 5 7 8^ Benefits of the microTNE-guidance include avoiding general anaesthesia and related costs.^3 5^ However, previous reports have contained few patients and no comparisons to conventional transoral TEE-guided procedures except on image quality. The aim of this study was to compare the feasibility, safety and time needed for microTNE and TEE guided PFO closures.

## Methods

### Study population

Consecutive patients (n=364) signed for percutaneous PFO closure in a tertiary centre, heart and lung centre, Helsinki University Hospital (HUS), from 1 January 2003 to 16 November 2021 were included in the study. American Heart Association and European Society of Cardiology guidelines on PFO closures were followed.^2 9^ Indications for PFO closure comprised stroke (n=326), embolic acute myocardial infarction (n=1), peripheral embolism (n=2) and decompression sickness (n=5) (table 1). Patients (n=7) who had other closure indications (dyspnoea, stress desaturation) or an ASD (n=10) were excluded.

**Table 1:** Baseline variables of the study population (n=336) with hypothesis testing based on procedure type

	microTNE-patients (n=169)	TEE-patients (n=167)	P value
Age, years (SD)	46.6 (9.6)	45.8 (10.7)	0.47
Female, n (%)	60 (35.5)	70 (41.9)	0.26
BMI, kg/m^2^ (SD)	25.9 (4.0)	26.6 (3.9)	0.16
BMI>30, n (%)	20 (11.8)	23 (13.8)	0.14
History of smoking, n (%)	53 (31.4)	58 (34.7)	0.30
Family history of stroke, n (%)	43 (25.4)	47 (28.1)	0.39
Migraine, n (%)	76 (45.0)	73 (43.7)	1.0
Hypertension, n (%)	24 (14.2)	31 (18.6)	0.30
Hypercholesterolaemia, n (%)	40 (23.7)	16 (9.6)	<0.001
Diabetes mellitus, n (%)	4 (2.4)	4 (2.4)	1.0
RoPE score (SD)	7 (1.4)	7 (1.5)	0.002
Medication before closure, n (%)			
Acetylsalicylic acid (ASA)	7 (4.1)	17 (10.2)	0.03
Warfarin	1 (0.6)	1 (0.6)	1.0
ASA+dipyridamole	6 (3.6)	7 (4.2)	0.78
Dipyridamole	1 (0.6)	1 (0.6)	1.0
Clopidogrel	2 (1.2)	5 (3.0)	0.28
ACE inhibitor	4 (2.4)	12 (7.2)	0.04
Angiotensin II receptor blocker	15 (8.9)	17 (10.2)	0.71
Calcium channel blocker	2 (1.2)	10 (6.0)	0.02
Beta blocker	5 (3.0)	8 (4.8)	0.41
Diuretic	0 (0.0)	4 (2.4)	0.06
Statin	17 (10.1)	24 (14.4)	0.25
Antidiabetic medication	2 (1.2)	3 (1.8)	0.68
Stroke, n (%)	165 (97.6)	162 (97.0)	0.75
TIA, n (%)	26 (15.4)	35 (21.0)	0.20
Embolic AMI, n (%)	0 (0.0)	1 (0.6)	0.50
Peripheral embolism, n (%)	0 (0.0)	2 (1.2)	0.25
Decompression sickness, n (%)	4 (2.4)	1 (0.6)	0.37
Infarction in imaging, n (%)	161 (95.3)	156 (93.4)	0.49
MRI, n (%)	153 (90.5)	148 (88.6)	0.60
Infarction, n (%)	149 (97.4)	143 (96.6)	0.52
CT, n (%)	119 (70.4)	125 (74.9)	0.39
Infarction, n (%)	71 (59.7)	82 (65.6)	0.23
TTE, n (%)	165 (97.6)	156 (93.4)	0.07
LVEF, % (SD)	62.7 (7.1)	63.5 (6.5)	0.29
LVEDD, mm (SD)	48.6 (5.3)	49.3 (4.7)	0.36
PFO, n (%)	10 (5.9)	9 (5.8)	1.0
TEE, n (%)	166 (98.2)	162 (97.0)	0.50
PFO, n (%)	134 (79.3)	100 (61.7)	<0.001
Shunt, n (%)	74 (43.8)	67 (41.4)	0.51
Bubble contrast, n (%)	159 (94.1)	132 (81.5)	<0.001
Positive bubble contrast, % (n)	157 (98.7)	120 (90.9)	0.002
Floppy or aneurysmatic septum, n (%)	57 (33.7)	43 (26.5)	0.12

Variables are presented as mean and SD or frequency and percentage.

AMI, acute myocardial infarction; BMI, body mass index; LVEDD, left ventricular end-diastolic diameter; LVEF, left ventricular ejection fraction; microTNE, transnasal echocardiography with microprobe; PFO, patent foramen ovale; RoPE, Risk of Paradoxical Embolism; TEE, transoesophageal echocardiography; TIA, transient ischaemic attack; TTE, transthoracic echocardiography.

All patients were examined by neurologists to exclude other causes of embolisation than a PFO. Conventional TEE was used to diagnose the PFO and a significant right-to-left shunt.^2 10^ Additionally, the anatomy of the interatrial septum and PFO were visualised with TEE.

MicroTNE was introduced for PFO closures in November 2018 at the HUS. The study population was divided into two according to the procedure type: microTNE-group and TEE-group. PFO closure was successful with 336 patients. Additionally, closure was attempted but could not be performed due to not finding the PFO tunnel (n=9), a too large PFO in relation to the size of the atrium for any device (n=1) or the patient developing a coronary thrombosis during closure (n=1). These 11 cases were considered as closure failures and excluded from the study.

**Figure 1:**
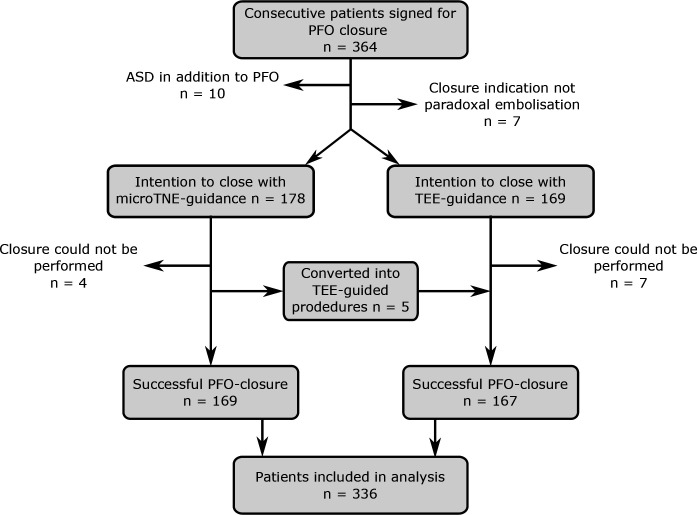
Flow chart of study patients. Initially, 364 consecutive patients signed for PFO closure were included in the study. Twenty-eight patients were excluded from analyses based on outlined exclusion criteria. Image processing: Inkscape. ASD, atrial septal defect; microTNE, transnasal echocardiography with microprobe; PFO, patent foramen ovale; TEE, transoesophageal echocardiography.

In the early adoption phase of the microTNE-technique, there was a learning curve with five patients converted to TEE-guided procedures. With experience, there were no more TEE conversions after the first 38 patients. Four closures were performed using the microTEE-probe orally because of narrow nasal anatomy. These patients were included in the microTNE-group. In total, 28 patients were excluded from the analyses (figure 1). Thus, 169 microTNE-patients and 167 TEE-patients were included in the analyses.

The patients underwent clinical evaluation and echocardiography the next day before discharge. Body temperatures were measured on the procedure day morning and the morning after. Blood tests were drawn 1–2 weeks before and the morning after PFO closure. These included C reactive protein (CRP) and leucocyte count. Patients were followed for 3 months after the procedure. ECG and transthoracic echocardiography were performed 3 months after closure.

### Procedures

MicroTEE-probe (S8-3t-probe, Philips, Amsterdam, Netherlands) was used in the microTNE procedures. As the probe has only 32 ultrasound elements, it has limited resolution and lacks three-dimensional capabilities. For microTNE procedures, patients were presedated with intravenous fentanyl (100 µg) and midazolam (1–2 mg) boluses. Local anaesthesia with lidocaine–adrenaline (20 mg/12.5 µg to each nostril) was administered to the nasal cavities. Topical anaesthesia with lidocaine spray (10 mg/spray, 10 sprays) was applied to the oral cavity to reduce probe sensation during the procedure. Next, the microTEE-probe was introduced through a nostril, as previously described.^11^ All procedures with conventional TEE were performed under general anaesthesia and intubation.

The PFO closure was performed similarly in both microTNE and TEE-guided procedures. After achieving an adequate image of the interatrial septum, an ultrasound-guided femoral venous access was introduced. PFO size was measured with balloon sizing under X-ray fluoroscopy. The closure device was then advanced through the PFO and deployed at each side of the septum. Before releasing the device, its adequate positioning was verified with echocardiography and fluoroscopy. Procedure times were recorded. Routine single antiplatelet therapy with acetylsalicylic acid or clopidogrel was applied indefinitely according to European Society of Cardiology recommendations.^2^

### Statistics

Statistical analyses were performed with SPSS (V.28, IBM). Normally distributed data were compared using the two-tailed t-test after Levene’s test. Non-normally distributed data were analysed using the Mann-Whitney U test and categorical data with the Fisher’s exact test. Correlations between variables were analysed using Spearman’s r. A logistic regression model was used to classify CRP increase. In all analyses, a p<0.05 and in correlation tests |R|>0.30 were considered significant.

## Results

Baseline characteristics of the study population are shown in table 1. The study population consisted of 206 men and 130 women, aged from 17 to 73 years. PFO closure was guided either with microTNE (n=169) or TEE (n=167).

Closure success rate was 97.7% with microTNE guidance and 96.0% with TEE guidance (p=0.54). PFO size was measured with balloon sizing in 92.3% of patients. Balloon diameter under X-ray fluoroscopy was 7.8±2.2 mm in the microTNE group and 9.1±3.4 mm in the TEE group (p<0.001). Most closures were performed using an 18 or 25 mm Amplatzer PFO Occluder. The procedure time was 21±7 min with microTNE and 30±13 min with TEE-guided closures (p<0.001) and the total time in the operating room was 66±15 min and 87±20 min (p<0.001), respectively. There was no difference in fluoroscopy time between the cohorts with an exposure time 4.8±3.0 in microTEE and 4.8±3.7 (min/procedure; mean±SD) in TEE guidance (p=ns). Procedure time and the total time (including anaesthesia or sedation) correlated with the CRP level after closure (R=0.33 and R=0.36, respectively) but not with temperature or leucocyte level after the procedure.

**Figure 2:**
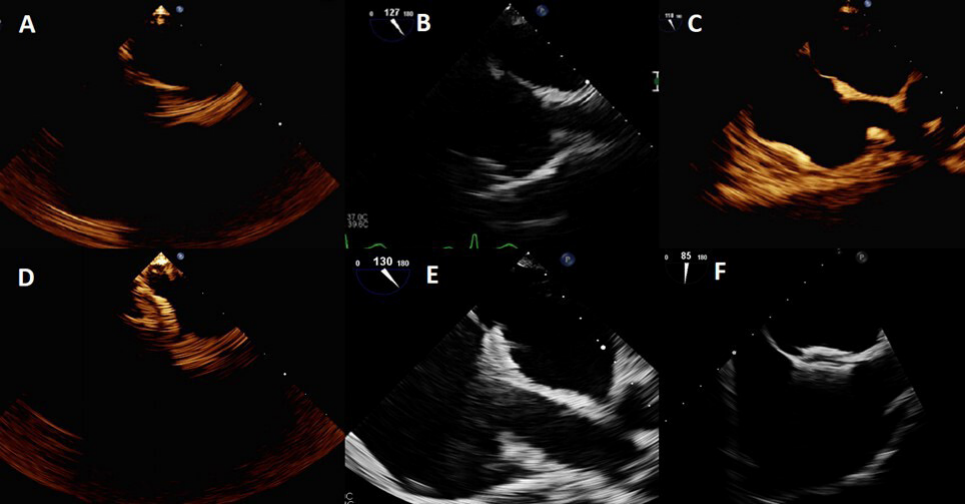
MicroTEE-probe image quality. The image quality of transoesophageal echocardiography with the microTEE-probe varied from poor (A, D) to acceptable (B, E) and good (C, F). (A–C) Bicaval images taken at the beginning of the procedure. (D–F) Acquired at the end of the procedure with the PFO occlude device in its final position. A poor image quality was characterised by blurry structures and large pixels while some structures were not visible at all (A, D). The image quality was good enough for device implantation, but detailed analyses was not possible. With acceptable image quality (B, E) most structures were visible, and structures in the near field could be studied with some details. A good image quality (C, F) was characterised by clear structure borders and resolution of the details near ordinary transoesophageal probe. PFO, patent foramen ovale; TEE, transoesophageal echocardiography.

**Figure 3:**
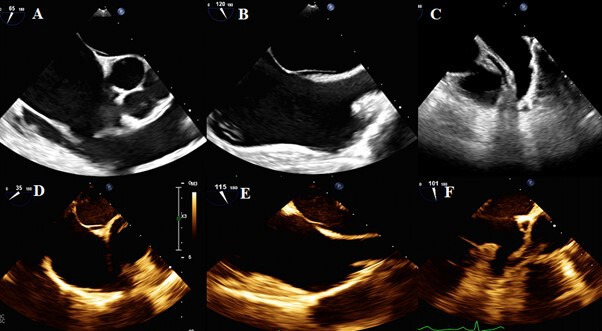
Comparison between conventional and microTEE-probe image quality. The diagnostic images (A–C) were acquired with Philips X8-2t TEE-probe. The interventional images of the same patient (D–F) were acquired with Philips microTEE-probe S8-3t. Even though the image quality with the microTEE probe was good, a smaller pixel size and more detailed images can be appreciated in A–C compared with D–F. (A, D) A short axis image of the atrial septum, (B, E) Bicaval view of the atrial septum and (C, F) left atrial appendix. TEE, transoesophageal echocardiography.

The image quality with microTEE-probe varied from poor to good, being poor in 20 (12%) patients, acceptable in 78 (47%) patients and good in 68 (41%) patients. In two patients, images were not stored, and therefore, could not be evaluated (figure 2). We did not do comparison on the same patients between conventional TEE and microTEE, because it would be difficult to do the imaging simultaneously and unethical sequentially. However, comparison in different patients could be appreciated in figure 3.

Adverse events during closure and follow-up are summarised in table 2. Two patients in the TEE group suffered from anaesthesia-related complications, which included an anaphylactic reaction to an anaesthetic and momentarily waking during anaesthesia. In the microTNE group, the procedure was interrupted in five cases either due to epistaxis (n=2), narrow nasal anatomy (n=2), mucus (n=1) or discomfort (n=1). These procedures were completed with conventional TEE under general anaesthesia on another occasion. These problems were concentrated in the first 38 patients treated using microTNE. Afterwards, the narrow nasal anatomy was circumvented in four cases by inserting the microTNE-probe transorally. Overall, the microTNE-route was successful in 97.2% of patients, whereas the route success rate in the TEE-group was 100.0% (p=0.06). The transnasal route was successful in 95.5% of cases.

**Table 2:** Adverse events during PFO closure and follow-up with hypothesis testing based on procedure type

	microTNE (n=169)	TEE (n=167)	P value
Patients with adverse events during follow-up, n (%)	38 (22.5)	42 (25.1)	0.61
Anaesthesia-related complications	0 (0.0)	2 (1.2)	0.25
Atrial fibrillation during closure	2 (1.2)	2 (1.2)	1.0
Atrial fibrillation during follow-up	7 (4.1)	4 (2.4)	0.54
RBBB	1 (0.6)	0 (0.0)	1.0
First-degree atrioventricular block	1 (0.6)	0 (0.0)	1.0
Closure device embolisation	1 (0.6)	0 (0.0)	1.0
Access site complication	5 (3.0)	12 (7.2)	0.09
Nasal complication	10 (5.9)	0 (0.0)	0.002
Presyncope or syncope	8 (4.7)	5 (3.0)	0.57
Chest pain or dyspnoea	7 (4.1)	9 (5.4)	0.62
Migraine worsening	1 (0.6)	12 (7.2)	0.001
Stroke	0 (0.0)	0 (0.0)	NA
Transient ischaemic attack	1 (0.6)	0 (0.0)	1.0
Deceased	0 (0.0)	1 (0.6)	0.50

Variables presented as frequency and percentage.

MicroTNE, transnasal echocardiography with microprobe; PFO, patent foramen ovale; RBBB, right bundle branch block; TEE, transoesophageal echocardiography.

**Figure 4:**
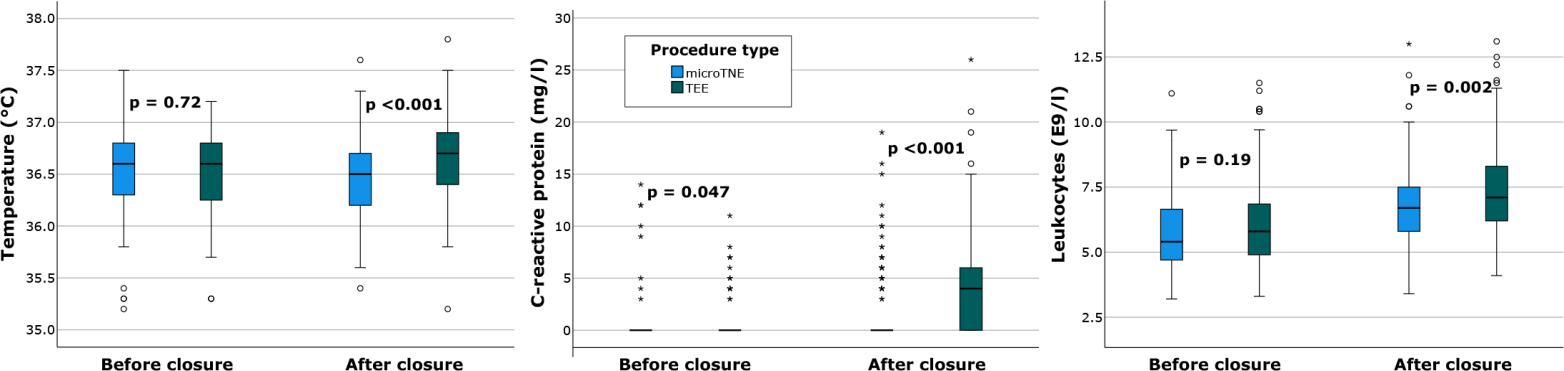
Temperature, C reactive protein (CRP) and leucocytes before and after PFO closure in both procedure types with Mann-Whitney U test. After PFO closure all inflammatory values were higher in the TEE group. There was also a statistically significant difference in CRP before closure. Notably, the clinical significance of this finding is likely low, as the differences consist of a small number of outliers and most patients had immeasurably low CRP before closure. Image processing: SPSS, Inkscape. MicroTNE, transnasal echocardiography with microprobe; PFO, patent foramen ovale; TEE, transoesophageal echocardiography.

The findings on inflammatory reactions are summarised in table 3. Temperature, CRP and leucocytes were significantly higher in the TEE group than in the microTNE group the morning after closure. Inflammatory reaction measured by temperature elevation or CRP change before procedure versus after procedure was significantly milder with microTNE than TEE (figure 4).

**Table 3:** Temperature, CRP and leucocytes with hypothesis testing based on procedure type

	microTNE (n=169)	TEE (n=167)	P value
Temperature, °C (SD)			
Procedure day morning	36.5 (0.43)	36.5 (0.47)	0.72
Morning after closure	36.5 (0.39)	36.7 (0.39)	<0.001
Change	−0.1 (0.4)	0.2 (0.6)	0.001
Fever (>37.5°C), n (%)			
Procedure day morning	0 (0.0)	0 (0.0)	NA
Morning after closure	1 (0.6)	1 (0.6)	1.0
CRP, mg/L (SD)*			
Before closure†	0 (2.0)	0 (1.8)	0.047
Day after closure	0 (3.5)	4 (4.5)	<0.001
Change	1 (2.9)	3 (4.0)	<0.001
CRP≥4 mg/L, n (%)			
Before closure†	7 (4.1)	16 (9.6)	0.054
Day after closure	36 (21.3)	84 (50.3)	<0.001
Leucocytes, E9/L (SD)			
Before closure†	5.8 (1.5)	6.0 (1.7)	0.19
Day after closure	6.8 (1.5)	7.3 (1.7)	0.002
Change	1.0 (1.3)	1.3 (1.7)	0.08
Leucocytes >8.2E9/L, n (%)			
Before closure†	10 (5.9)	17 (10.2)	0.16
Day after closure	21 (12.4)	44 (26.3)	0.001

Variables are presented as mean and SD or frequency and percentage.

*CRP<4=0.

†1–2 weeks before closure.

CRP, C reactive protein; microTNE, transnasal echocardiography with microprobe; NA, not applicable; TEE, transoesophageal echocardiography.

Nasal complications occurred only in the microTNE group: The procedure provoked epistaxis in nine patients (5.3%) four of which (2.4%) required treatment. Two patients were treated with a haemostatic sponge and two required an ear, nose and throat specialist. One patient experienced nasal congestion for 2 months after the procedure. Seventeen patients had an access site complication (n=5 in the microTNE group, n=12 in the TEE group, p=0.09, six patients had more than one of these complications) consisting of inguinal resistances (n=14), notable inguinal haematomas (n=8), inguinal pseudoaneurysms (n=2) and a retroperitoneal haematoma (n=1). Bleeding complications were equally common in both groups (p=0.84).

Epistaxis was not associated with an inflammatory response after closure (p>0.05 for all). Access site complications and history of smoking increased leucocytes after closure in both groups. However, significant between-group differences in leucocyte levels remained, when data were stratified by access site complication or history of smoking (p=0.008 and p<0.001, respectively). Female gender was associated with a higher temperature and lower leucocyte levels after closure compared with males (p=0.001 and p<0.001, respectively). Again, when data were stratified by gender, it did not account for the differences between microTNE and TEE (p<0.05 for all). Hypertension and the use of medications such as angiotensin II receptor blockers or calcium channel blockers were associated with higher CRP levels after closure, but they did not explain the differences between the groups either (p<0.001 for all). A logistic regression model to classify increased CRP levels showed significant independent association with procedural time (B=0.001, p=0.003) and procedure type (B=0.87, p=0.004) but no association with age, hypertension, BMI or suffering a complication during follow-up. Overall, no variables from table 1 nor adverse events from table 2 explained the differences in inflammatory markers between the groups.

There was one device embolisation in the microTNE group, which occurred 4 days after closure. Migraine worsening was reported less often in the microTNE group than the TEE-group (n=1 vs n=12, respectively, p=0.001). No patients suffered a recurrent stroke. However, one patient in the microTNE group had a TIA and one patient in the TEE-group died of unknown reasons 2 months after PFO closure.

## Discussion

The clinical motive to adopt microTNE into PFO procedures in our clinic was limited anaesthesia resources. With few published reports on microTNE-guided PFO closures, there were feasibility and safety concerns. This study confirms microTNE feasibility with an equally high success rate after a learning curve, shorter procedure times and milder inflammatory reactions than conventional procedures with general anaesthesia.

### MicroTNE versus TEE

In general, microTNE has been found a feasible and safe alternative to conventional TEE.^3 4 7 12^ However, the resolution is lower and image quality worse, but deemed adequate.^7 12–14^ This corresponds to our experience, with higher patient-to-patient variation in image quality compared with conventional TEE. However, image quality did not prevent procedures being completed.

Previous reports indicate a high success rate of microTNE-insertion at 84%–96%,^4 7 13 14^ equal to our transnasal route success rate. Reported problems with the nasal route include narrow nasal passage, not getting the probe into the oesophagus and intolerable pain.^4 7 13 14^ Initial endotracheal malpositioning occurred in 5% of patients in one study.^14^

MicroTNE is performed under local anaesthesia, local vasoconstrictor and mild sedation.^13^ Patients tolerate microTNE well in 72%–98% of cases with significant gagging or discomfort occurring in 2%–6%.^4 7 13^ Minor nasal bleeding has been reported in 0%–31% of procedures,^4 7 13 14^ but few patients (0%–2%) need treatment.^4 14^ In our study, nine patients (5.3%) developed epistaxis during or after the procedure with four (2.4%) needing treatment. Most cases of epistaxis occurred during the learning curve of the procedure.

A hypothetical problem with microTNE is a bigger risk for thermal injury due to the ultrasound energy concentrating on a smaller surface and being used at its maximum.^14 15^ In practice, different angles and views are used reducing the risk on an individual area of mucosa. Due to the smaller size, greater angulation and thus potentially higher likelihood to become fixed in extreme flexion, the pressure caused by the microTNE-probe on mucosa may be greater than that of a conventional TEE-probe, which might increase the risk of perforation.^14 15^ There are no publications on these issues.

Another hypothetical problem with microTNE might be bacteraemia caused by nasal injury during probe insertion, but it has not been a clinically significant issue.^14^ To the knowledge of the authors, bacteraemia after microTNE has not been investigated. In one study, the incidence of bacteraemia after conventional TEE was 1.4%.^16^

Two small studies have published results on microTNE-guided PFO closure.^3 5^ Greco *et al* studied 20 patients who underwent PFO closure without complications, and 6 months after closure no residual shunts were detected.^3^ Klettas *et al* found the image quality of microTNE to be adequate compared with conventional TEE during structural cardiac interventions which included few (n=12) PFO and ASD closures.^5^

Traditionally, transoral TEE has been used to guide PFO closure, but it requires general anaesthesia with intubation.^5 6 17^ The main advantage of microTNE-guided PFO closure is that no general anaesthesia is needed.^3 5 7 13^ Sedation and intubation increase procedure duration, risks and costs.^3–6^ The need for general anaesthesia could also be removed by using intracardiac echocardiography (ICE), but this increases intracardiac manipulation and cost of the procedure due to requiring single-use ICE catheters.^5 6 17^ The use of microTNE shortens procedural duration and makes postprocedural monitoring unnecessary.^3^ Consequently, more patients can be treated daily, which reduces procedure queues. Once the use of microTNE becomes established, there is no need for an anaesthesia team during the procedure improving resource allocation.

### TEE safety

Though conventional TEE is considered a safe and semi-invasive method, severe, even life-threatening complications have been reported.^18–20^ Potential TEE-related complications include lip bruising or laceration (13%), dental trauma (0.03%–0.1%), hoarseness (10%–12%), accidental tracheal insertion (0.02%), oropharyngeal, oesophageal or gastric laceration or perforation (<0.01%–0.3%), major pharyngeal bleeding (<0.01%–0.8%), thermal injury or burn and tongue necrosis.^14 18 20^ Overall, TEE-related complication rate has been estimated to be 0.18%–2.8% and TEE-related mortality in non-operative instances <0.01%–0.02%0.^18^ Reported rates of major intraoperative TEE-related complications are similar to non-operative ones and have been estimated to be 0.1%–1.2%.^18 20^

Most patients (86%) have been found to develop an oesophageal or gastric injury after conventional TEE used during structural cardiac interventions.^21^ These varied from intramural haematomas and mucosal lacerations to petechiae and ecchymosis, with the haematomas and lacerations accounting for 47% of injuries.^21^ Longer procedural time under TEE and poor image quality are associated with these injuries.^21^

### General anaesthesia and inflammation

General anaesthetics are widely believed to cause perioperative hypothermia and attenuate fever by impairing thermoregulatory control.^22 23^ Postoperatively, fever is more likely due to surgical trauma, possible infection or allergy and mismatched blood transfusions.^23^ There is no evidence that general anaesthesia itself would induce fever.

Surgical trauma and other invasive procedures as well as mechanical ventilation, stress and pain are known to influence inflammatory responses.^24^ Anaesthetic agents used in general anaesthesia have also been discovered to have complex immunomodulating effects.^24^ Most anaesthetic agents have been found to suppress the inflammatory response by directly disrupting immune cell functions and indirectly modulating the stress response.^24^ Further, opioids, used frequently in anaesthesia, inhibit the immune system.^24^ In clinical settings, it can be difficult to distinguish the effect of anaesthesia on the immune response from the effects of other factors such as surgical trauma.^24^

The greater probability of fever and higher CRP and leucocyte levels the day after PFO closure in the TEE group can be the result of general anaesthesia or greater trauma caused by the TEE-probe. As addressed previously, general anaesthesia has not been found to cause fever or elevated inflammatory markers in earlier studies. Thus, the larger TEE-probe may cause more trauma than the smaller microTNE-probe thereby provoking a more distinct inflammatory response. The clinical relevance of this remains indeterminate but may indicate that even a short procedure using the TEE-probe can cause pharyngeal and oesophageal trauma.

### Transoral microTEE

Several centres have reported using microTEE transorally to guide left atrial ablation as well as left atrial appendage, ASD and PFO closure procedures.^25–27^ This is a feasible alternative, but it carries a risk of probe damage by biting and induces more laryngeal discomfort than the nasal route. However, in cases of narrow nasal anatomy, we have begun to use the transoral route as an alternative with otherwise equal procedure flow.

### Strengths and limitations

The patients included in the two groups of this single-centre, non-randomised study, were treated during different time periods. Thus, the indications for PFO closure changed, which can be seen in measured PFO sizes. However, we had access to patients’ complete clinical data. Due to microTNE-guided PFO closure being a new technique in our clinic, nasal route-related complications were more common early after the technique’s introduction. This might have affected the total number of microTNE-related complications but gives a realistic picture of the associated learning curve. Further, CRP, leucocytes and temperatures were only recorded from 96.1%, 98.0% and 69.0% of patients, respectively.

## Conclusion

This study shows that a microprobe echocardiography inserted via the nasal route is a feasible and safe alternative to conventional TEE in guiding PFO closure. Using microTNE decreases procedure duration since no general anaesthesia is needed and induces a weaker inflammatory response than conventional TEE. Furthermore, microTNE is well tolerated and provides adequate image quality for PFO closure. The wider adoption of microTNE for this procedure can provide reduced procedural costs and increased efficiency.

## Impact on daily practice

PFO closure guided by microTNE can be tolerated well under conscious sedation and is a feasible and safe alternative to conventional TEE in guiding PFO closure. PFO closure guided with microTNE has an equally high success rate, shorter procedural duration with no general anaesthesia needed and, unexpectedly, induces a weaker inflammatory response than conventional TEE. Furthermore, microTNE provides adequate image quality for PFO closure. The wider adoption of microTNE guidance for this procedure can reduce the need for already limited anaesthesia resources and increase efficiency.

## Data Availability

Data are available on reasonable request.

## References

[1] Hagen PT, Scholz DG, Edwards WD. Incidence and size of patent foramen ovale during the first 10 decades of life: an autopsy study of 965 normal hearts. Mayo Clin Proc 1984;59:17–20. 10.1016/s0025-6196(12)60336-x 6694427

[2] Pristipino C, Sievert H, D’Ascenzo F, et al. European position paper on the management of patients with patent foramen ovale. General approach and left circulation thromboembolism. Eur Heart J 2019;40:3182–95. 10.1093/eurheartj/ehy649 30358849

[3] Greco C, Chiavari PA, Campolongo G, et al. Transnasal transesophageal echocardiography: a new approach for the PFO occlusion in awake patients. Catheter Cardiovasc Interv 2008;72:538–41. 10.1002/ccd.21665 18814237

[4] Fukuda S, Shimada K, Kawasaki T, et al. Transnasal transesophageal echocardiography in the detection of left atrial thrombus. J Cardiol 2009;54:425–31. 10.1016/j.jjcc.2009.07.002 19944318

[5] Klettas D, Alcock E, Dworakowski R, et al. Is transnasal TEE imaging a viable alternative to conventional TEE during structural cardiac interventions to avoid general anaesthesia? A pilot comparison study of image quality. Echo Res Pract 2017;4:1–7. 10.1530/ERP-16-0029 28249937 PMC5435876

[6] Boccalandro F, Baptista E, Muench A, et al. Comparison of intracardiac echocardiography versus transesophageal echocardiography guidance for percutaneous transcatheter closure of atrial septal defect. Am J Cardiol 2004;93:437–40. 10.1016/j.amjcard.2003.10.037 14969617

[7] Spencer KT, Goldman M, Cholley B, et al. Multicenter experience using a new prototype transnasal transesophageal echocardiography probe. Echocardiography 1999;16:811–7. 10.1111/j.1540-8175.1999.tb00133.x 11175225

[8] Wang B, Zhang L, Sun W, et al. Transnasal transesophageal echocardiography guidance for percutaneous left atrial appendage closure. Ann Thorac Surg 2019;108:e161–4. 10.1016/j.athoracsur.2019.01.039 30807735

[9] Slottow TLP, Steinberg DH, Waksman R. Overview of the 2007 Food and Drug Administration circulatory system devices panel meeting on patent foramen ovale closure devices. Circulation 2007;116:677–82. 10.1161/CIRCULATIONAHA.107.709972 17679629

[10] Giblett JP, Williams LK, Kyranis S, et al. Patent foramen ovale closure: state of the art. Interv Cardiol 2020;15:e15. 10.15420/icr.2019.27 33318751 PMC7726850

[11] Spencer KT, Krauss D, Thurn J, et al. Transnasal transesophageal echocardiography. J Am Soc Echocardiogr 1997;10:728–37. 10.1016/s0894-7317(97)70116-0 9339424

[12] Ferns S, Komarlu R, Van Bergen A, et al. Transesophageal echocardiography in critically ill acute postoperative infants: comparison of AcuNav intracardiac echocardiographic and microTEE miniaturized transducers. J Am Soc Echocardiogr 2012;25:874–81. 10.1016/j.echo.2012.05.009 22749435

[13] Zimmermann P, Greim C, Trautner H, et al. Echocardiographic monitoring during induction of general anesthesia with a miniaturized esophageal probe. Anesth Analg 2003;96:21–7. 10.1097/00000539-200301000-00006 12505917

[14] Greim CA, Brederlau J, Kraus I, et al. Transnasal transesophageal echocardiography: a modified application mode for cardiac examination in ventilated patients. Anesth Analg 1999;88:306–11. 10.1097/00000539-199902000-00015 9972746

[15] Urbanowicz JH, Kernoff RS, Oppenheim G, et al. Transesophageal echocardiography and its potential for esophageal damage. Anesthesiology 1990;72:40–3. 10.1097/00000542-199001000-00008 2297132

[16] Mentec H, Vignon P, Terré S, et al. Frequency of bacteremia associated with transesophageal echocardiography in intensive care unit patients: a prospective study of 139 patients. Crit Care Med 1995;23:1194–9. 10.1097/00003246-199507000-00007 7600826

[17] Hijazi Z, Wang Z, Cao Q, et al. Transcatheter closure of atrial septal defects and patent foramen ovale under intracardiac echocardiographic guidance: feasibility and comparison with transesophageal echocardiography. Catheter Cardiovasc Interv 2001;52:194–9. 10.1002/1522-726x(200102)52:2<194::aid-ccd1046>3.0.co;2-4 11170327

[18] Hilberath JN, Oakes DA, Shernan SK, et al. Safety of transesophageal echocardiography. J Am Soc Echocardiogr 2010;23:1115–27; 10.1016/j.echo.2010.08.013 20864313

[19] Sainathan S, Andaz S. A systematic review of transesophageal echocardiography-induced esophageal perforation. Echocardiography 2013;30:977–83. 10.1111/echo.12290 23834425

[20] Piercy M, McNicol L, Dinh DT, et al. Major complications related to the use of transesophageal echocardiography in cardiac surgery. J Cardiothorac Vasc Anesth 2009;23:62–5. 10.1053/j.jvca.2008.09.014 19058977

[21] Freitas-Ferraz AB, Bernier M, Vaillancourt R, et al. Safety of transesophageal echocardiography to guide structural cardiac interventions. J Am Coll Cardiol 2020;75:3164–73. 10.1016/j.jacc.2020.04.069 32586591

[22] Lenhardt R. The effect of anesthesia on body temperature control. Front Biosci (Schol Ed) 2010;2:1145–54. 10.2741/s123 20515846

[23] Sessler DI. Perioperative thermoregulation and heat balance. Lancet 2016;387:2655–64. 10.1016/S0140-6736(15)00981-2 26775126

[24] Rossaint J, Zarbock A. Perioperative inflammation and its modulation by anesthetics. Anesth Analg 2018;126:1058–67. 10.1213/ANE.0000000000002484 28922235

[25] Snijder RJR, Renes LE, Swaans MJ, et al. Microtransesophageal echocardiographic guidance during percutaneous Interatrial septal closure without general anaesthesia. J Interv Cardiol 2020;2020:1462140. 10.1155/2020/1462140 32982607 PMC7492935

[26] Barreiro-Perez M, Cruz-González I, Moreno-Samos JC, et al. Safety, and utility of microtransesophageal echocardiography guidance for percutaneous LAAO under conscious sedation. JACC Cardiovasc Interv 2019;12:1091–3. 10.1016/j.jcin.2019.02.027 31171287

[27] Stec S, Zaborska B, Sikora-Frac M, et al. First experience with microprobe transoesophageal echocardiography in non-sedated adults undergoing atrial fibrillation ablation: feasibility study and comparison with intracardiac echocardiography. Europace 2011;13:51–6. 10.1093/europace/euq349 20880953

